# Related Structures in the Straight Sinus: An Endoscopic Anatomy and Histological Study

**DOI:** 10.3389/fnana.2020.573217

**Published:** 2020-10-29

**Authors:** Yuanliang Ye, Jiuyang Ding, Shaoming Huang, Qiujing Wang

**Affiliations:** ^1^The National Key Clinical Specialty, Guangdong Provincial Key Laboratory on Brain Function Repair and Regeneration, The Engineering Technology Research Center of Education Ministry of China, Department of Neurosurgery, Zhujiang Hospital, Southern Medical University, Guangzhou, China; ^2^Department of Neurosurgery, Liuzhou General Hospital, Liuzhou, China; ^3^School of Forensic Medicine, Guizhou Medical University, Guiyang, China; ^4^Department of Anatomy, Guangxi Medical University, Nanning, China; ^5^Department of Cerebrovascular Surgery, The Third Affiliated Hospital, Sun Yat-Sen University, Guangzhou, China

**Keywords:** straight sinus, great cerebral vein, endoscopy, chordae willisii, anatomy

## Abstract

Some structure might be encountered with endovascular procedures within the straight sinus and is not now readily seen on digital subtraction angiography (DSA). We investigated the morphological and histological characteristics of the straight sinus, chordae willisii (CW), and junction between the great cerebral vein (GCV) and straight sinus. A total of 22 cadaveric heads and 135 patients were analyzed with either anatomic dissection or neuroimaging. The morphological features of the CW and the junction between the GCV and straight sinus were analyzed by endoscope. The histology of the junction between the GCV and straight sinus was evaluated under the microscope with staining for elastic fiber, Masson’s, and immunohistochemistry. We found that fold, elevation, small bugle, or nodule and CW were detected by endoscope in the straight sinus. The most common type of CW was valve-like lamellae, which comprised 40.46% of all CW. Three different types of junctions between the GCV and straight sinus were identified: type 1 has folds in the GCV and elevation on the floor of the straight sinus; type 2 has folds and a small bugle; and type 3 presents with an intraluminal nodule located at the opening of the GCV. Compared with arachnoid granulation, the nodule consists of smooth muscle fibers and higher rate of elastic fibers. Understanding the detailed anatomy of the straight sinus may help surgeons to avoid procedural difficulties and to achieve higher success rate.

## Introduction

The straight sinus, which receives the venous blood from the cerebellar, thalamus, and basal ganglia and terminates into the confluence of the sinuses, is the most important venous drainage in the deep venous systems of the cranium. Although the transvenous approach is the first-line therapeutic strategy in treating the deep brain vascular diseases involving the straight sinus (Gomez et al., 2000; Stam et al., 2008; De Beritto et al., 2015; Pu et al., 2017), there is much more to know about its histology, morphology, and intraluminal structures.

Imaging and standard anatomical methods have already been used to describe the anatomical structure of the straight sinus (Saxena et al., 1974; Browder et al., 1976; Shin et al., 2000; Tsutsumi et al., 2018; Kim et al., 2019). For instance, using ultrastructural analysis, histological knowledge of the straight sinus composition and the possibility of straight sinus constituent layers dissection have been well illuminated (Amato et al., 2016). The junction between the great cerebral vein (GCV) and straight sinus, which is located in the rostral part of the straight sinus, presents an elevation in the bottom of the straight sinus (Ghali et al., 1989). Some literatures have well described the anatomy of this junction by macroscopic methods (Dagain et al., 2008). However, the chordae willisii (CW) in the straight sinus have not been studied in detail so far. Moreover, the nodule at the junction was recognized as arachnoid granulations, and its histological structure has not been described.

In the present study, we performed a high-definition rigid endoscopy to observe the morphological features of the CW and junction between the GCV and straight sinus. Furthermore, we investigated the histological characteristics of the CW and nodules with elastic fiber staining, Masson’s staining, and immunohistochemistry, aiming to provide anatomical basis for endovascular procedures within the straight sinus.

## Materials and Methods

A total of 22 anatomical specimens, which were obtained from fresh autopsy in the Department of Anatomy of Guangxi Medical University, were fixed in 10% formalin solution for 4–8 weeks. All specimens were from patients older than 18 years old. The mean age of the specimens was 62 ± 13.19 (41–79) years, including 9 male and 13 female patients. The exclusion criteria were: (1) craniocerebral trauma; (2) neurological disease; and (3) disease affecting the dura sinuses. All patients or their families’ members signed an individual informed consent to approve the use of samples. This study was carried out in accordance with the recommendations of the ethics committee of Guangxi Medical University.

### Endoscope Assessment

The scalps were removed with special attention to expose the inion. The cranial vault (calvaria) above the axial plane across the nasion and the inion was removed by a surgical power device (Xishan, China). Then, the brain tissue around the straight sinus was en bloc resected, whereas the brain tissue around the junction between the GCV and straight sinus was removed underneath the pia mater in piecemeal fashion. The straight sinus was flushed with tap water to remove blood clots. Then, a high-definition rigid endoscope (Karl Storz, Germany) measuring 2.7 mm in diameter and optics (0 and 30°) was inserted into the lumen of the sinus from torcula herophili. Special attention was given to the morphology of the CW and junction between the GCV and straight sinus.

### Dissecting Microscope Assessment

The GCV, partial inferior sagittal sinus, and straight sinus were carefully removed en bloc under a surgical microscope (OPMI6, Zeiss). The superior sagittal sinus (SSS) and right and left transverse sinus (TS) were transversely sectioned 3 cm from the torcula herophili. The observation area included the anterior SS, posterior straight sinus, and GCV.

### Light Microscope Assessment

Five venous sinus samples containing nodules at the junction between the GCV and straight sinus were prepared for microscopic assessment. Hematoxylin and eosin (H&E) staining, Masson’s trichrome staining (for detecting collagen fibers), and Victoria blue staining (for detecting elastic fibers) were applied to sectioned samples. To differentiate the nodules from the arachnoid granules, 10 arachnoid granulations in the SSS were also subjected to H&E, Masson’s trichrome, and Victoria blue staining. The histological sections were analyzed using a Zeiss Axioskop plus microscope (Carl Zeiss Microscopy) at ×50, ×100, and ×400 magnifications. Images were acquired and stored by the AxioVision software. For elastic fibers quantitation, a series of five equally spaced tissue sections (~0.5 mm apart) spanning the entire nodes and arachnoid granules was stained with Victoria blue. The elastic fibers quantification was determined as the % of blue staining area in three randomly chosen fields (×400) using the ImageJ software. All samples were conducted in a blind manner.

### Immunohistochemistry Analysis

A specific antibody against the N-terminal portion of SMA-a protein (mouse anti-human SMA-a monoclonal antibody) was used in an immunohistochemical study on samples containing the nodule. Paraffin embedded samples were cut into 4 μm thick successive coronal sections. After deparaffinization and dehydration in serial alcohol concentrations (70–100%), the endogenous peroxidase activity was blocked for 30 min in methanol containing 0.3% hydrogen peroxide. Moreover, 5% bovine serum albumin (BSA) was used for avoiding nonspecific binding of antibodies. Antigen retrieval was performed in target retrieval solution for 30 min. Sections were then incubated with primary antibody diluted 1:100 for 30 min at room temperature. A horseradish peroxidase-labeled secondary antibody was applied for 30 min at room temperature after the primary antibody.

### MRI-Enhanced Analysis

There were 135 patients enrolled in the study, including 59 male and 76 female patients. The mean age at diagnosis was 50.63 ± 15.23 years (range: 27–76 years). The indications for MRI-enhanced analysis were suspicion of an intracranial lesion (48 patients) and evaluation of cerebral vascular diseases (65 patients) or tumor (22 patients). Exclusion criteria were: (1) cerebral vascular diseases involved with the SS and GCV; (2) intracranial tumor involved with the pineal region, third ventricle, and corpus callosum; and (3) the GCV and straight sinus were unclear in the imaging. MRI images were obtained as previously described (Kim et al., 2019).

### Statistical Analysis

All statistical analyses were performed using SPSS 22 for Windows (SPSS Inc., Chicago, IL, USA). Categorical data, including numbers and percentages of CW, were summarized using descriptive statistics. Numerical data were expressed as means, SDs, minimums, and maximums. Independent-sample Student’s *t*-test was used to compare the distribution of elastic fibers in the nodules and that in the arachnoid granulation. One-way ANOVA with Tukey’s *post hoc* test was used to examine the difference in diameter among the GCV, GCV–SS, and SS.

## Results

### Chordae Willisii in the Straight Sinus

#### Endoscopic Observations

**Figure 1:**
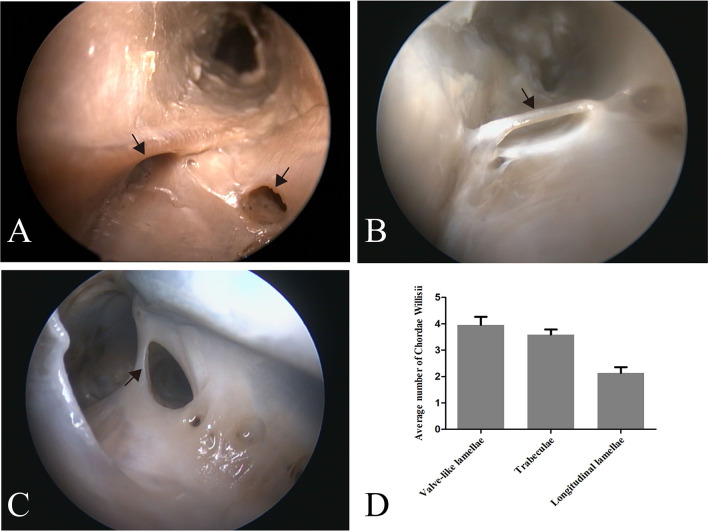
Endoscopic view of chordae willisii (CW) in the straight sinus. A valve-like CW located at the junction between the lateral and inferior wall [arrow in panel **(A)**]. A trabecula in the lumen of the SS [arrow in panel **(B)**]. A longitudinal chord inside the sinus, which divided the lumen into two channels [arrow in panel **(C)**]. Graphs showing comparisons of the number of valve-like lamellae, trabeculae, and longitudinal lamellae in the straight sinus **(D)**.

There were 215 counts of CW in 22 examined straight sinuses, averaging 9.78 CW per straight sinus. The most number of intraluminal structures in the straight sinus was 14, whereas the lowest was 6. Details of CW were given in Table 1 and Figure 1D.

**Table 1:** Findings in all cadaveric straight sinus specimens and the number of chordae willisii (CW) in the straight sinus by endoscopic assessment.

Specimen no.	Chordae willisii		
	Valve-like lamellae	Trabeculae	Longitudinal lamellae
1	6	4	1
2	6	3	1
3	3	5	2
4	2	3	3
5	2	3	4
6	3	3	3
7	3	5	1
8	4	3	2
9	3	5	3
10	5	3	1
11	6	4	4
12	5	3	3
13	6	3	2
14	2	4	3
15	6	4	1
16	4	3	2
17	3	5	1
18	4	3	2
19	2	5	1
20	5	3	3
21	3	2	1
22	4	3	3

Eighty-seven valve-like lamellae were found in our study, contributing to 33.85% of the CW in the examined sinuses. Most valve-like lamellae were found in the posterior portion of the straight sinus at the junction between the lateral and inferior wall of the sinus (Figure 1A). The trabecula, which made up 30.74% of all CW in our study, was the second most common form of CW in the straight sinus. The trabeculae could be identified either centrally or peripherally in the lumen (Figure 1B). The laminar chord, which comprised 18.29% of all CW, represented the least form of CW (Figure 1C).

#### Distribution of CW in the SS

The valve-like lamellae and longitudinal lamellae were found predominantly in the posterior portion of the straight sinus (*t* = 5.34, *p* = 0.000; *t* = 5.16, *p* = 0.000). The trabeculae were found in the anterior and posterior portions of the SS (*t* = 0.44, *p* = 0.663).

#### Histological Analysis

**Figure 2:**
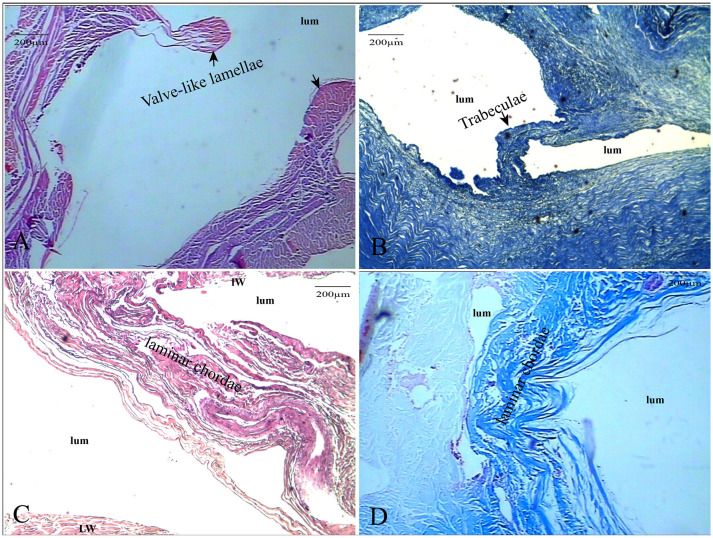
The CW were composed of collagen fibers and endothelial cells. The valve-like CW [arrows in panel **(A)**] between the lateral sinus and lumen (HE, ×100); the valve-like type [arrows in panel **(B)**] present at the inferior wall of the vein (HE, ×100); lamellar cord [arrows in panels **(C,D)**] divided the sinus into different sizes of lumens (HE and Masson’s staining, ×100).

HE staining, elastic fiber staining, and collagen fiber staining were used to observe the histological characteristics of the CW in the straight sinus. The CW were composed of collagen fibers and endothelial cells arranged in different ways. The diameter of the valvular fibrous cord was small, which separates the dura sinus from the lumen (Figure 2A). The lamellar fibrous cord divides the sinus into different sizes of lumens (Figure 2B), and the trabecular fiber connects the bottom wall and side wall of the straight sinus (Figures 2C,D).

### The Junction Between the Great Cerebral Vein and the Straight Sinus

#### Endoscopic Observations

**Figure 3:**
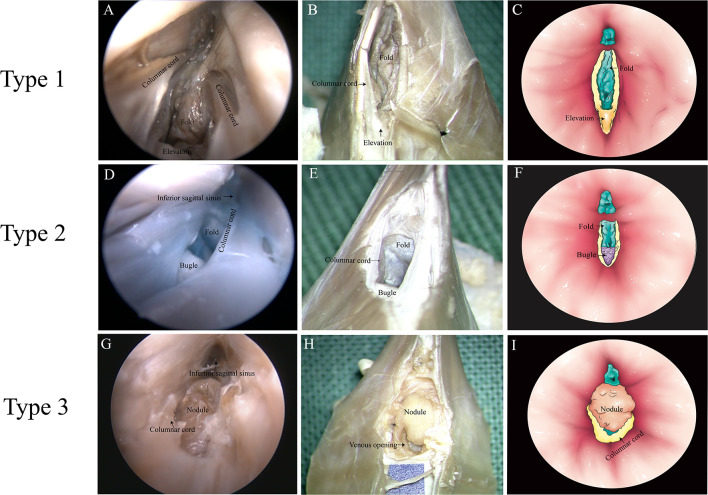
Types of junction between the great cerebral vein (GCV) and the straight sinus. **(A,D,G)** Endoscopic view. **(B,E,H)** Microscopic dissection view. **(C,F,I)** Topographic schematic illustration.

There were three anatomic types of junctions found (Table 2). Type 1: valve-like fold in the GCV with an elevation on the floor of the SS. Around the elevation, there were columnar cords on each side of the SS wall in 12 cases of this study (Figures 3A–C). Type 2: valve-like fold on the posterior wall in the GCV with a small bugle that bellowed columnar cords. This anatomic type was observed in four cases (Figures 3D–F). Type 3: an intraluminal nodule of variable sizes situated at the opening of the GCV with extended columnar cord on the horizontal plane. This intraluminal nodule partially blocked the lumen of the straight sinus. This anatomic type was identified in six cases (Figures 3G–I).

**Table 2:** Different anatomic types in the junction between the great cerebral vein and SS.

Type	Anatomic characteristics
1	The valve-like fold was presented in the great cerebral vein and an elevation in the floor of the SS.
2	The valve-like fold was also observed in the great cerebral vein. A small bugle was located on the posterior wall of the great cerebral vein.
3	An intraluminal nodule was situated on the opening of the great cerebral vein and exceeded the horizontal plane of the columnar cord.

#### Imaging Analysis

**Figure 4:**
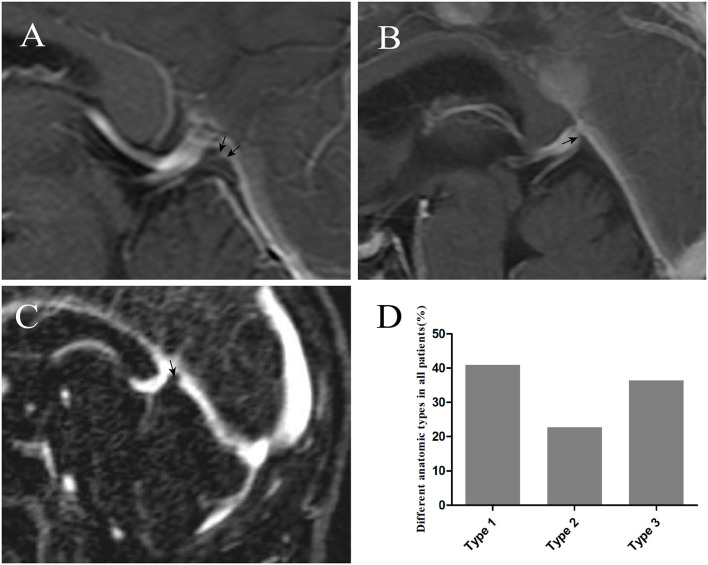
The injection of iodinated contrast material helped delineate elevation [arrow in panel **(A)**], small bugle [arrow in panel **(B)**], and nodule [arrow in panel **(C)**] from normal cerebral tissues. Graphs showing comparisons of the number of type 1, type 2, and type 3 at the junction **(D)**.

With the injection of iodinated contrast material, the three anatomic types (elevation, small bugle, and nodule) at the junction between the GCV and straight sinus were delineated from normal cerebral tissues (Figures 4A–D). Types 1, 2, and 3 made up 40.74%, 22.96%, and 35.56% of all patients, respectively. Moreover, the diameters in the GCV, GCV–SS, and straight sinus were 4.27 ± 0.80, 3.74 ± 1.10, and 5.03 ± 1.40, respectively, with statistical significance (*p* = 0.000; Table 3).

**Table 3:** General data of the 135 patients with MRI scan.

Variables	M ± SD/%
Mean age (years)	50.63 ± 15.23
Male, no. (%)	59 (43.70)
**Diagnosis, no. (%)**
Suspicion of an intracranial lesion	48 (35.56)
Evaluation of vascular diseases	65 (48.15)
Tumor	22 (16.30)
**Type of the junction, no. (%)**
Type 1	55 (40.74)
Type 2	31 (22.96)
Type 3	48 (35.56)
**Diameter of the junction (mm)**
GCV	4.27 ± 0.80
GCV–SS	3.74 ± 1.10
SS	5.03 ± 1.40

#### Morphological Comparison of the Nodule, Arachnoid Granulation, and SS

**Figure 5:**
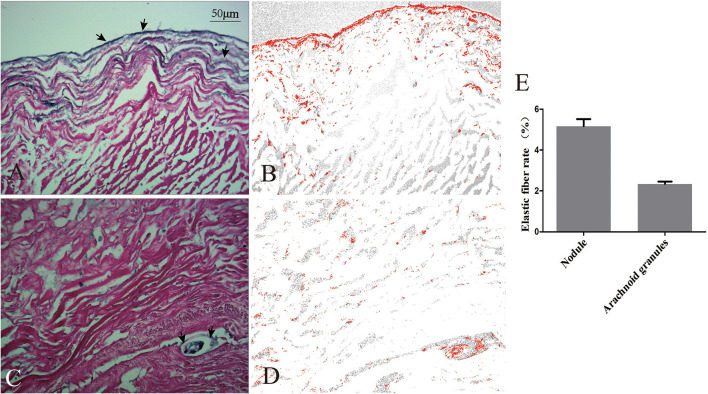
Comparison of elastic fibers between the nodule and arachnoid granulation. Elastic fibers (arrows) were demonstrated on the surface of the nodule **(A)** and scattered in the arachnoid granulation **(C)**. The areas occupied by elastic fibers were measured *via* software **(B,D)**. Graphs showing comparisons of the rate of elastic fibers between the nodule and arachnoid granulation **(E)**.

The nodule was composed of collagen and surrounded on the surface by elastic fibers (Figures 5A,B). The arachnoid granules were made up of collagen fibers, connective tissue, and arachnoid cells (Figures 5C,D). The percentage of elastic fibers was higher in the nodule than in the arachnoid granules (Figure 5E).

#### Immunohistochemistry Analysis of the Nodule

**Figure 6:**
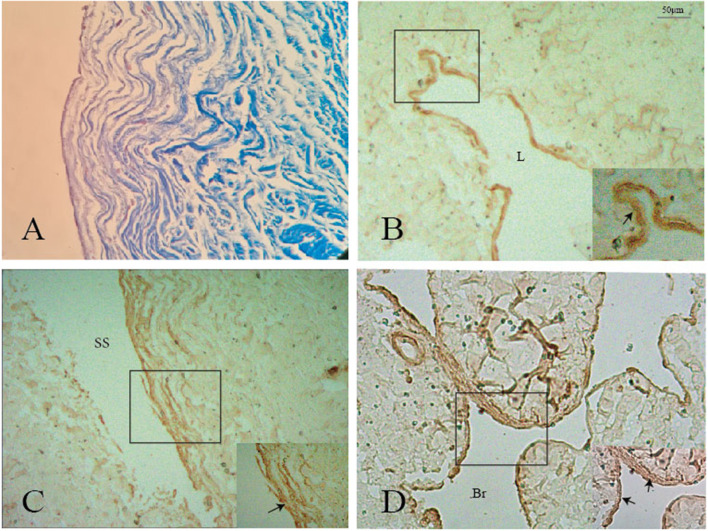
Positive staining of smooth muscle (black arrows) was shown on the surface of the nodule **(A,B)**, GCV **(C)**, and its branch **(D)**. L: lumen of the great cerebral vein. Br: branch of the great cerebral vein.

Immunohistochemistry analysis showed that SMA was expressed in the surface of the nodule and intersection between the GCV and sinus walls (Figures 6A–D), but was not expressed in the lateral sinus wall.

## Discussion

This study has demonstrated the presence of CW in the straight sinus. The valve-like lamellae were the most common form and mainly distributed in the posterior portion of the straight sinus. Fold, elevation, small bugle, and nodule were found at the junction between the GCV and the straight sinus. Unlike arachnoid granules, the nodule was composed of collagen fibers, elastic fibers, and smooth muscles.

### Chordae Willisii

Neurosurgeons have increasingly turned their attention to the CW that were recognized by early anatomists. Schmutz stated in detail about the morphological characteristics of CW in the SSS and divided the CW into three different forms: valve-like lamellae, longitudinal lamellae, and trabeculae (Schmutz, 1980). Some researchers confirmed cord-like structures in the lumen of cerebral veins ostia and networks of accessory cords in the venous lacunae (Sharifi et al., 2004; Shao et al., 2009). Similar to our data, valve-like lamellae was the most common type of CW in the straight sinus. Different from the bridge veins that emptied into the SSS, the bridge veins around the tentorium cerebelli, which collect venous drainage of the cerebellum, occipital lobe, and temporal lobe, flowed into the tentorial sinus and then entered into the straight sinus or TS (Matsushima et al., 1989; Muthukumar and Palaniappan, 1998; Rosenblum et al., 2019). This pattern of venous reflux revealed that there was no direct connection between the bridge vein and the straight sinus, and the valve-like lamellae might just be a drainage channel in the SS to guarantee a laminar venous blood confluence in the sinuses. The longitudinal chords were mostly detected at the opening of the straight sinus. They divided the SS into double or triple lumens (Kaplan et al., 1972; Kaplan and Browder, 1976), which increased flow velocity and led to a decrease of static pressure at the orifices.

**Figure 7:**
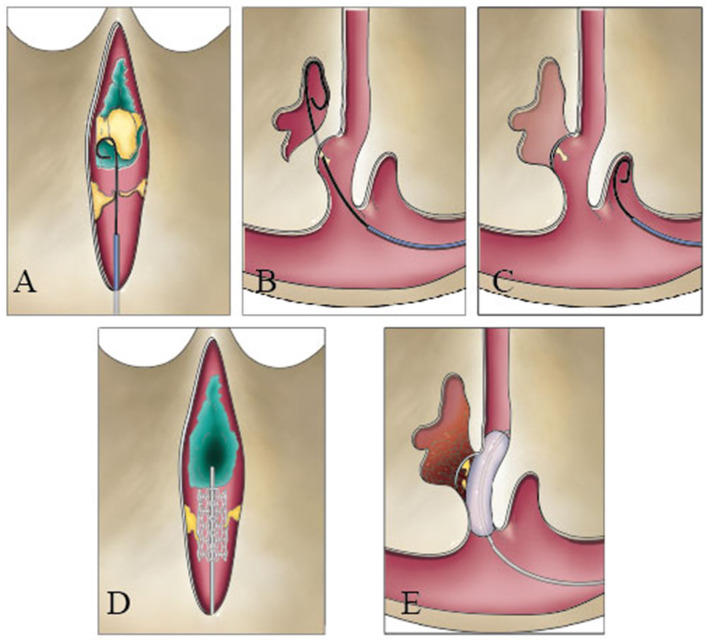
Catheter could be blocked during a transvenous endovascular approach in the straight sinus. The guide wire could hinder the nodule **(A)**, pass through the valve-like cord **(B)**, and run inward in blind cavity **(C)**. The stent could damage the fibrous cord **(D)**, or the balloon squeezes the thrombus into the lateral sinus or bridge vein **(E)**.

The knowledge of intraluminal structures helps the interventional neuroradiologists to navigate microfilaments and catheters through the dura sinus (Farb, 2007; Iwanaga et al., 2020). The existence of valve-like lamellae and longitudinal lamellae in the straight sinus could cause the catheter moving into a blind cavity in the posterior portion of the straight sinus. A catheter could progress into the narrow channels surrounded by the trabeculae and the sinus wall and cause the stent to open unsuccessfully (Figures 7A–E). Therefore, understanding the detailed anatomy of the straight sinus may help surgeons to avoid procedural difficulties and to achieve higher success rate.

### Junction Between the Great Cerebral Vein and the Straight Sinus

The physiological regulation of the deep cerebral venous system has not been well understood. What is more, few researchers have paid attention to the anatomy of the junction between the GCV and the straight sinus. Velut described a fold of the GCV and the origin of the junctions in the straight sinus and assumed that the fold would create a pouch between the venous wall and the dura (Velut, 1987). Ghali et al. (1989) reported the presence of a body and a small elevation at the junction, which had a smooth surface and convex upwards with dimensions ranging between 3.5–4.5 mm in length and 2.5–3.0 mm in width at the basal part. In the present study, under the endoscope, we categorized the junction into three types: type 1 junction has folds in the GCV and has an elevation on the floor of the SS; type 2 has folds and bulges at the junction; and type 3 has a nodule and extends on the horizontal plane of the cord.

MRI can clearly define normal intraluminal architecture within the dura venous sinuses. Arachnoid granulations were considered well-defined, hypointense, or isointense on T1- and hyperintense on T2-weighted sequences and the nature of the intra-sinus filling defects images (Farb, 2007). Haroun et al. (2007) showed a 75-year-old female patient with the presence of rounded, well-defined intraluminal structure at the junction of the straight sinus and the vein of Galen. In our study, the elevation, small bugle, or nodule was revealed by MRI-enhanced analysis. The fold was not presented for blood flow in the junction. Based on the anatomic types of junctions, type 1 was the most common in the general population.

A risk of hemorrhage existed if the microwire/microcatheter catches on the origin of veins while maneuvering along in the deep venous system. Based on the types of junction, the tip of the microcatheter can be steam shape as an arc or S-shape with different angles. Shaped catheters aid access to branches at angles and provide a stable position once placed in the venous cavity without contacting the venous wall. In addition, the GCV–SS was easy to tear in type 2 for thin organizational structure on the posterior wall of the GCV during thrombectomy in the SS. Moreover, the anatomic types of junction mean different physiological regulations of venous blood flow in the GCV–SS.

### Histological Analysis of the Nodule

Traditionally, the arachnoid granulation tissue could form a nodule that appeared as a ball-shaped valve preventing the venous return from the third and lateral ventricles and effecting the secretion of the cerebrospinal fluid. Unlike an arachnoid granulation that contains collagen fibers and a few amounts of the elastic fiber, we found that the nodule contains elastic fiber and smooth muscles in addition to collagen fibers. Since smooth muscles were observed at the intersection between the GCV and the wall of the straight sinus, the smooth muscles of the nodule probably had the same histological origin as the GCV. The existence of elastic fibers and smooth muscles was to maintain the physiological regulation of venous blood flow in the straight sinus. The smooth muscle in the GCV could increase GCV pressure; meanwhile, the smooth muscle contracts to reduce the diameter of the nodule and thus reduce its volume, and finally, the venous blood flows into the straight sinus through their combination.

The nodule and SS have similar histological structure. All the nodule and SS exhibited an endothelial cell, a thin layer of subendothelial connective tissue containing collagen and elastic fibers, and the presence of a muscle layer. In our study, the collagen fibers in the nodule were connected with the inferior wall of the SS, with the same course. Elastic fibers were also demonstrated on the surface of the SS, which connected with that of the nodule.

### Embryogenesis of the Intraluminal Structures

In embryonic development, the intraluminal structures in the dura sinuses probably come from the cells of the primitive veins in the epidural space, which merges to form different sized venous sinuses. During the formation of the SS, folds may form since the brain tissue is compressed between the mesencephalon and rhombencephalon (Senders et al., 2018). Accompanying the formation of these folds, the anterior and posterior plexuses combine to form the various venous sinuses. Based on the differences in the forms of fusion among different individuals, many variations, including position in the TC, channel duplication, and CW, appeared in the subsequent sinuses (Kaplan and Browder, 1976).

## Limitations

We acknowledge that our study has some limitations. First, cadaveric heads vascular replica imperfectly reflects the flexibility of intracranial vessels. Second, it does not provide guidance about how to avoid intraoperative damage to the intraluminal structures during transvenous endovascular therapy.

## Conclusion

We verified the presence of CW in the straight sinus and illustrated the morphological structures of the junction between the GCV and the straight sinus from the endoscopic standpoint and histological observation. The knowledge of the internal structures of the straight sinus provides a foundation for surgeons to navigate through technical problems.

## Data Availability Statement

The data that support the findings of this study are available from the corresponding author upon reasonable request.

## Ethics Statement

The studies involving human participants were reviewed and approved by ethics committee of Guangxi medical university. The patients/participants provided their written informed consent to participate in this study.

## Author Contributions

QW, YY, and SH: conception and design. YY and JD: acquisition of data and illustration. YY and SH: analysis, interpretation of data and statistical analysis. YY: drafting the article. All authors: critical revision of the manuscript and manuscript review. QW: manuscript final version approval on behalf of all authors. QW: study supervision. All authors contributed to the article and approved the submitted version.

## Conflict of Interest

The authors declare that the research was conducted in the absence of any commercial or financial relationships that could be construed as a potential conflict of interest.

## Abbreviations

TS: transverse sinus
CW: chordae willisii
Br: branch of the great cerebral vein
GCV: great cerebral vein
IW: inferior wall
LW: lateral wall.
